# DREADDs: The Power of the Lock, the Weakness of the Key. Favoring the Pursuit of Specific Conditions Rather than Specific Ligands

**DOI:** 10.1523/ENEURO.0171-19.2019

**Published:** 2019-10-11

**Authors:** Raphaël Goutaudier, Véronique Coizet, Carole Carcenac, Sebastien Carnicella

**Affiliations:** Grenoble Institut Des Neurosciences, Institut National de la Santé et de la Recherche Médicale, U1216, Université Grenoble Alpes, 38000 Grenoble, France

**Keywords:** behavior, C21, clozapine, CNO, DREADDs, electrophysiology

## Abstract

**Figure:**
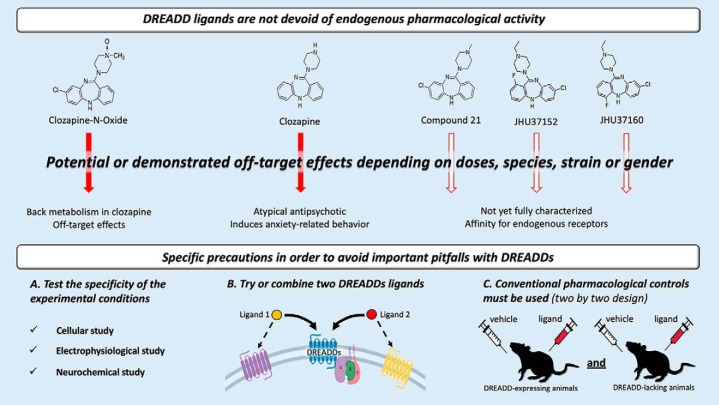

## Significance Statement

DREADDs (designer receptors exclusively activated by designer drugs) are a powerful and tremendous new technique for selectively manipulating a specific neuronal (or non-neuronal) subpopulation. Recent studies indicate, however, that ligands used for DREADDs, such as clozapine-*N*-oxide or its parent compound clozapine, are not as selective as expected, even at reasonable concentrations. Although the new generation of ligands specifically developed for DREADDs or alternative chemogenetic receptors may present some improvements, the absence of potential off-target effects remains to be fully demonstrated. Together, indications from the recent literature on DREADDs should warn current and future users about some weaknesses of this expanding technique in the field of integrative neuroscience and encourage them to take some specific precautions to avoid important pitfalls with DREADDs, which remain a promising and complementary approach to optogenetics with the relevant controls.

## 

Over the past decade, chemogenetic and optogenetic techniques have revolutionized integrative neuroscience by providing new tools to reversibly manipulate the activity of specific populations or neurotransmitter systems with greater selectivity (Sternson and Roth, 2014; Roth, 2016; Wiegert et al., 2017). Compared with optogenetics, which allow fast and phasic neuronal modulation with high temporal resolution, chemogenetics allow more extended modulation of systems, which is particularly useful for studies focusing on tonic phenomena (e.g., investigation of the implication of dopamine in motivational processes; Whissell et al., 2016). Among chemogenetic tools, designer receptors exclusively activated by designer drugs (DREADDs) are widely used and are referred to as a biological “lock-and-key” system for selective manipulation of cell activity through G-protein signaling pathways. First developed very elegantly by the Roth’s group (Armbruster et al., 2007), this G-protein-coupled receptor (GPCR) is a muscarinic receptor: the lock, which was mutated to respond only to clozapine-*N*-oxide (CNO), the key, a derived metabolite of the atypical antipsychotic clozapine, otherwise with, potentially, no pharmacological activity.

However, since 2016, some publications have raised worrying issues concerning the use of CNO. First, relatively high doses of CNO (10 mg/kg) administered systemically can have pharmacological off-target activity, as evidenced by the induction of behavioral effects in rats and mice not mediated by DREADDs (MacLaren et al., 2016; Gomez et al., 2017; Baerentzen et al., 2019)_._ In addition, Gomez et al. (2017) showed that CNO does not readily cross the blood–brain barrier, may exhibit low DREADD binding affinity, and was back-metabolized into clozapine, becoming the real effector of the DREADDs. Based on these striking observations, they suggested the direct use of low doses of clozapine (0.1 mg/kg) to activate DREADDs instead of CNO. However, using low doses of clozapine instead of large doses of CNO gradually converting to clozapine leads to two major limitations. First, it is not obvious that clozapine, in acute injection or prolonged diffusion, acts on DREADDs in the same way (Mahler and Aston-Jones, 2018). Second, since clozapine is an atypical antipsychotic agent, it has numerous endogenous targets such as serotoninergic, muscarinic, or dopaminergic receptors, with relatively strong affinities (Meltzer, 1989; Schotte et al., 1993; Brunello et al., 1995; Ashby and Wang, 1996; Armbruster et al., 2007) and are likely to induce off-target effects, even with low doses. Indeed, the 0.1 mg/kg dose of clozapine recommended for DREADD experiments has been found to significantly increase anxiety-related behavior in mice (Manzaneque et al., 2002) as well as in rats (an effect that we also observed; R. Goutaudier and S. Carnicella, unpublished observations), where clozapine also affects locomotion through potential sedation and impairs cognitive flexibility (Ilg et al., 2018).

Although the occurrence of these effects may depend on the species, strain, or sex used, and may be very discrete (as not all behavioral dimensions are affected); they have the potential to significantly interfere with the performance of animals in a variety of behavioral tasks. High stress and anxiety can be confounding factors in memory or pain studies, for example (Sousa et al., 2006; Sorge et al., 2014). Moreover, behavior related to drugs of abuse or psychiatric disorders such as schizophrenia, anxiety, or cognitive flexibility can modify the whole phenotype (Floresco et al., 2009; Koob and Schulkin, 2018). Based on these observations, important precautions are needed with clozapine to avoid biased behavioral studies.

Would a new molecule specifically designed for DREADDs be more selective? Compound 21 (C21) is a synthetic DREADD ligand, developed in 2015 (Chen et al., 2015) and partially characterized *in vitro* as well as *in vivo* in 2018 (Thompson et al., 2018). Once again, at low doses (<3 mg/kg), it was described to be devoid of behavioral off-target effects and able to alter the behavior of DREADD-expressing animals. This study was strengthened by Jendryka et al. (2019), who conducted pharmacokinetic and pharmacodynamic experiments using mice and C21 (3 mg/kg). They showed that 30 min after C21 administration the concentration of the molecule in the CSF was >10 times higher than the estimated EC_50_ for DREADD activation ([C21]_CSF_ = 40 nm and EC_50DREADDs_ = 3 nm), and without back-metabolization into clozapine. However, the results of a recent *BioRxiv* preprint study in rats, mice and macaques (Bonaventura et al., 2018) suggest that, although C21 exhibits a low brain penetrance, a dose of 1 mg/kg may already modify brain function in wild-type mice. In addition, a weak affinity and occupancy for DREADDs was observed *in vitro* in rat brain slices, as well as *in vivo* in mice and macaques in a positron emission tomography study. Based on the same experimental investigation as for C21, they proposed, as an alternative, two other ligands, JHU37152 and JHU37160, that possess a higher *in vivo* potency for DREADDs and potentially fewer off-target effects (Bonaventura et al., 2018). Although this new generation of DREADD ligands appears promising, due to their novelty, they are still poorly characterized and remain structurally homologous to clozapine and CNO. Exhaustive characterization in cellular to behavioral investigation will therefore be crucial to exclude the potential pitfalls found for CNO.

An alternative solution for improving the selectivity of chemogenetic approaches would be to use another lock and key combination. As such, the κ-opioid receptor-DREADD (KORD) is a mutated inhibitory GPCR derived from human κ-opioid receptor (Vardy et al., 2015). Compared with classical DREADDs that bind clozapine, CNO, C21, or JHU compounds, KORD is engaged by salvinorin B, a drug-like metabolite of the KOR-selective agonist salvinorin A. Although this chemogenetic approach was elegantly used in combination with an activatory DREADD to create an “ON and OFF” system within the same neuronal population (Vardy et al., 2015, Aldrin-Kirk et al., 2016), it remains marginally used because it only reduces the neuronal activity over a short period of time (Aldrin-Kirk and Björklund, 2019). In addition, salvinorin B exhibits some affinity for endogenous KOR at high concentrations and has not yet benefited from in-depth characterization as it is currently performed for DREADD-related compounds (Roth, 2016). Replacing the GPCR by a mutated ion channel, another alternative designed receptor called ligand-gated ion channels (LGICs) is an option developed by the Stenson laboratory (Magnus et al., 2011). Compared with DREADDs, LGICs combine the ligand-binding domain of a mutated nicotinic receptor with the ion pore domain of another chosen receptor to create a chimeric ion channel. Similar to DREADDs, this hybrid channel is activated by a small agonist derived from quinuclidinyl benzamide, an α7 nicotinic acetylcholine receptor agonist, and allows ion exchange across the neuronal membrane. Among other limitations specific to this approach (Aldrin-Kirk and Björklund, 2019), it also shares with DREADDs the use of a pharmacological ligand that can potentially interact with endogenous receptors, depending on the experimental conditions. Finally, all these locks derive from endogenous receptors and, as such, fail to get rid of the limitations intrinsically linked to pharmacology.

**Figure 1.**
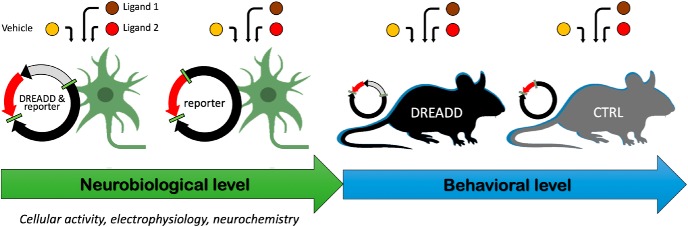
Three steps proposed to validate experimental conditions for DREADD selectivity and efficiency. (1) At a neurobiological level, find the optimal experimental conditions, ligand, and concentration with cellular, electrophysiological, or neurochemical experiments before behavioral investigation. (2) Confirm that an effect is DREADD mediated using two different ligands compared with the vehicle. (3) Do not forget to include DREADD-lacking animals (expressing only the reporter gene) during experiments to verify that the effect, at the chosen dose, is specific to the receptor–ligand interaction.

Beyond these recent developments and the questions that remain, a crucial question should be asked: will a totally selective and inert key ever be found? Probably not. It is essential to bear in mind that DREADDs are chemogenetic tools combining genetics and pharmacology. Although genetic approaches offer powerful control of the expression of the locks (i.e., DREADDs) in specific cell populations, or subpopulations with conditional approaches, they derive from endogenous GPCRs and, as such, are subject to the same limits as classical pharmacology for the key. It is therefore unlikely that molecules will be found that will exhibit high binding affinity for DREADDs without affinity for some of the numerous receptors that are already present in the brain and are closely related to DREADDs. For instance, clozapine has a very high affinity for DREADDs, but also for the serotoninergic receptor 5-HT2 (Ki = 10^−8^ for both; Armbruster et al., 2007; Gomez et al., 2017), and a high affinity for a broad range of other GPCRs (Ki = 10^−7^ to 10^−6^; Armbruster et al., 2007). Even synthetic ligands that are specifically designed for this chemogenetic technique exhibit substantial affinities for endogenous receptors. For instance, although JHU37152 and JHU37160 have a lower affinity for 5-HT receptors than clozapine, they have an overall similar target profile to this drug, with an even higher affinity for the muscarinic receptors (Bonaventura et al., 2018), suggesting potentially stronger off-target effects. C21 also exhibits a higher affinity for the histaminergic H1 receptor than for DREADDs (Ki > 10^−8^ and Ki = 10^−7.2^, respectively; Thompson et al., 2018) and a greater binding potential to opioid receptors than clozapine (Bonaventura et al., 2018). To minimize the pitfalls of this powerful approach, regardless of the key choice, critical precautions must be taken (Fig. 1).

First, before any behavioral experiment, the experimental conditions must be tested to find the best ligand and the optimal dose or doses, depending on the experimental approach. DREADDs must not be considered as a turnkey tool; cellular, neurochemical or electrophysiological experiments should be performed before the behavioral study (Mahler et al., 2014; Beloate et al., 2016; Boekhoudt et al., 2016) to confirm the efficacy of the ligand at the chosen dose in the system of interest. In addition, DREADD-lacking control animals must also be included to verify the absence of aspecific neurobiological effects of the ligand or the receptor per se that may be activated by a neurotransmitter or have a constitutive activity (Saloman et al., 2016).

Second, when possible, two different DREADD ligands should be tested to confirm that the observed behavioral effects are specifically DREADD mediated. The specific pharmacological actions on DREADDs would be similar, but off-target effects may differ.

Third, and the most critical point, conventional pharmacological controls must be used. The same philosophy as in pharmacology should be applied, and, as for neurobiological experiments, groups of transgenic animals without expressing DREADDs (e.g., DREADDs empty viral vectors) must be integrated to verify the selective effects of the ligand and chosen dose (Smith et al., 2016, Campbell and Marchant, 2018; Mahler and Aston-Jones, 2018; for an example of experimental studies following this design, see also Xia et al., 2017; Cope et al., 2019). This statement may appear trivial, but the pursuit of an absolute selective ligand and the attractiveness of this approach have already led to some overconfident behavioral studies, sometimes conducted in the complete absence of this control.

In conclusion, DREADDs provide a precise way of manipulating neural circuits and behavior, and afford a great alternative to optogenetics to tonically manipulate a specific cellular subpopulation, thus opening exciting new avenues of research. However, just as thermal properties of light in optogenetics can lead to aspecific effects (see also Owen et al., 2019), greater precaution is needed with chemogenetics, and standard controls must be mandatory. DREADD limitations must be recognized and time must be taken to avoid or to control possible off-target effects and to verify that this approach does not induce bias per se. Using the strengths of genetics while paying attention to the weaknesses of pharmacology will maximize the potential of this approach.

## Synthesis

Reviewing Editor: Kirill Martemyanov, The Scripps Research Institute Decisions are customarily a result of the Reviewing Editor and the peer reviewers coming together and discussing their recommendations until a consensus is reached. When revisions are invited, a fact-based synthesis statement explaining their decision and outlining what is needed to prepare a revision will be listed below. The following reviewer(s) agreed to reveal their identity: Bryan Roth.

The following points that require attention were identified during the discussion of the revised “commentary” manuscript.

1. The limitations, controls and appropriate experimental controls associated with the use of the DREADD technology have been discussed in the literature before. These reports (e.g. Roth, Neuron 2016 and others) need to be referenced in the appropriate context. Highlighting what specific issues that might have previously received limited attention are unique to this report would also help navigate the readers.

2. Please consider adding a cartoon or table illustrating/summarizing the 3 recommendations for the application of the chemogenetic approaches.

3. Confounds with JHU compounds (numerous potential off-target hits, similarity of pharmacology with clozapine) need to be discussed a bit more critically.

4. It will be helpful to include additional discussion on kappa opioid based and hybrid channel chemogenetic tools from Sternson lab (and their limitations) which should increase the profile and novelty of the commentary. Also, mentioning limitations of optogenetic approaches may be helpful to include (e.g. see paper from Kreitzer lab this year in Nature Neuroscience) to avoid creating an impression that chemogenetics is an inferior approach in general.
